# Transforming the Approach to Cell‐Based Therapies

**DOI:** 10.1002/sctm.17-0064

**Published:** 2017-04-29

**Authors:** Anthony Atala, Todd N. McAllister

Cell‐based therapies and the field of regenerative medicine have been heralded as the next pillar of medical care for more than 20 years. While public marrow and cord blood banking have been unequivocal successes, triumphs beyond blood diseases and cancer have been slow to arrive. With the passing of the 21st Century Cures Act, which was driven in large part by a desire to broaden the therapeutic reach of stem cells, it is an appropriate time to objectively review the clinical efficacy of stem cell therapies, and perhaps contemplate strategies to improve their clinical impact. Here, we discuss some of the underlying assumptions that have driven the field with respect to cell sourcing, mechanism of action, and immunoprivilege.

The debate on cell sourcing has been an enduring topic in regenerative medicine. While much of the attention originally focused on the ethical considerations associated with pluripotent embryonic stem cells, the vast majority of clinical trials have utilized multipotent stem cells, derived from either autologous marrow or fat, or allogeneic cells harvested from a young, healthy, master donor. The autologous stem cell approach has the advantage of being immunologically matched but is potentially limited by the fact that the cells are usually harvested from older patients, and their potency may be more limited. Autologous stem cell approaches also have the disadvantage of being difficult to standardize and scale, with quality assurance infrastructure hurdles that may limit widespread use. Because stem cells have relatively few surface antigens and do not trigger acute immune responses, allogeneic transplants provide an attractive alternative, with the presumed benefit of being sourced from younger, healthier donors. Improved manufacturing margins also make allogeneic approaches an appealing model from a commercial perspective. As allogeneic cells engraft and differentiate, however, they can begin to express surface markers that are recognized by the immune system. So, while allogeneic stem cells may provide a powerful, short‐term paracrine signal that may not trigger significant acute immune responses, their long‐term effect diminishes along with their immunoprivileged status.

With the benefit of nearly two decades of clinical hindsight using both autologous and allogeneic cells, three overarching conclusions are clear. First, stem cell injections are overwhelmingly safe. While there have been some isolated cases of ectopic tissue formation or teratoma formation, these complications have been extraordinarily rare. In fact, the most relevant risks associated with stem cell therapy are infection and stroke, which can generally be avoided with the proper manufacture and delivery of the cells. Second, long‐term cell engraftment and survival is extremely low, with most studies demonstrating significantly less than 1% cell engraftment at the target site. Third, while many studies have demonstrated promising results and encouraging trends, none have demonstrated dramatic improvements in pivotal trials.

The question, then, is what can be done to help give stem cell therapies the final push to achieve clear clinical impact? Some would argue that it is simply a matter of time to find the right combination of cell type, dosage, delivery strategy, and perhaps even the appropriate patient population. There is some evidence to support this “incremental progress” approach, as we have observed noteworthy successes in cartilage repair and graft‐versus‐host disease after more than 20 years of academic and clinical development and several failed clinical trials. It is puzzling, however, that despite tens of thousands of treatments across an exhaustive spectrum of clinical parameters, we have not been able to focus in on effective approaches to some of the main therapeutic targets for regenerative medicine (e.g., heart disease, stroke, and neurodegenerative diseases).

Over the last decade, there has been an increased emphasis on short‐term paracrine signaling as the primary mechanism of action for stem cell therapies. While it is clear that stem cells can release a milieu of growth factors and chemoattractants and can also play a role in immunomodulation, the field may have been too quick to abandon efforts to facilitate long‐term cell survival. It seems plausible that, by increasing cell survival and engraftment, we may prolong the paracrine signaling that triggers native healing mechanisms and may also provide differentiated cells capable of driving a functional repair. The conundrum, of course, is that neither high‐mileage autologous stem cells nor nonimmunomatched allogeneic cells are well suited to impart this prolonged therapeutic effect.

One potential solution to this conundrum is the use of banked cells collected from birth tissues, such as umbilical cord blood, umbilical tissue, or placenta. These cells have the advantage of being immunologically matched (using either a private autologous banking model or a public allogeneic banking model) and derived from young, healthy donors. Publicly banked cord blood has already made a profound impact in the reconstitution of bone marrow following chemotherapy, where long‐term engraftment and functionality is fundamental to the treatment. Somewhat surprisingly, the therapeutic targets of cord blood have been expanded beyond diseases of the blood, with FDA‐regulated trials exploring the use of cord blood‐derived hematopoietic stem cells to treat hypoplastic left heart syndrome, stroke, autism, and cerebral palsy. While these indications may benefit from the prolonged paracrine signal afforded by immunologically matched cord blood, other investigators are exploring the use of umbilical cord‐derived mesenchymal cells for indications requiring more complex tissue repair. In Asia, trials are ongoing for a broad range of indications, including cardiomyopathy, muscular dystrophy, spinal cord injury, amyotrophic lateral sclerosis, liver disease, and even type 1 diabetes. Early indications from these studies suggest not only solid safety profiles but also efficacy results that exceed the expectations of most in the field.

Despite complex scientific and regulatory challenges, stem cell therapies have made a steady march toward fulfilling their promise as the next pillar of medical care. With encouraging results across several indications, the field must now provide the final push in efficacy to justify making these therapies the standard of care. While we can argue the relative merits of traditional autologous and allogeneic stem cell models, or the contribution of paracrine signaling versus engraftment and functional repair, it seems likely that banked birth tissues will provide the best of both worlds. Ironically then, in the face of regulatory changes meant to provide a middle ground for minimally manipulated, point‐of‐care cell therapies, the emerging solution may be birth‐derived, banked cells.

